# Vital Statistics of Scott County, Ky., Digested from the Census as Taken in 1850

**Published:** 1851-07

**Authors:** W. L. Sutton

**Affiliations:** Georgetown, Ky.


ART. II.—*Vital Statistics of Scott county, Ky., digested from the census as taken in* 1850. By W. L. SUTTON, M. D., of Georgetown, Ky.

*The population of Scott county by the census of* 1840, *was*:

| | WHITES. | FREE BLACKS. | SLAVES. |
|---|---|---|---|
| Males, | 4,340 | 54 | 2,737 |
| Females, | 3,880 | 55 | 2,602 |
| | 8,220 | 109 | 5,339 |

*By census of* 1850, *it was*:

| | WHITES. | FREE BLACKS. | SLAVES. |
|---|---|---|---|
| Males, | 4,706 | 220 | 5,840 |
| Females, | 4,195 | | |
| | 8,901 | Total. | 14,961 |

Thus there has been an increase of whites by 681, or 8.28 per cent: of Free Blacks of 111, or more than 100 per cent: of Slaves, by 501, or something more than 9 per cent, in ten years.

Of the Whites there have been 158 deaths, or one in 56⅓, to-wit:

Of 4,706 males, 91 deaths, or 1 in 51.38

Of 4,195 females, 67 " or 1 in 61.12

Of Free blacks 220 in number, 2 deaths are returned, or 1 in 110*

---

*Both of these were adult males. I also know of two other adults one male, one female, free blacks, who died within the twelve months covered by the returns. It is likely that some children also died. If no children died the proportion of deaths among the whites and the free blacks was nearly equal.

Of 5,840 slaves, 154 deaths, or 1 in a fraction less than 38,

Of the whole populatlon, 314 deaths, or 1 in 47.66.

Of the whites, were born in Kentucky, 120, in Virginia 17, in Maryland 7, in Pennsylvania 5, in New England, England and Ireland, each 2, in Delaware, Missouri and Germany, each 1.

Of the Blacks, were born in Kentucky 142, in Virginia 12, in Maryland 2.

The deaths were arranged in months as follows:

*Winter.*—December 17, January 12, February 18—Total 47.

*Spring.*—March 25, April 29, May 24—Total 78.

*Summer.*—June 52, July 35, August 51—Total 138.

*Autumn.*—September 29, October 14, November 8—Total 51.

Of the 314 deaths, there were 158 Whites, 156 Blacks, 178 Males, 134 Females, 2 sex not stated.

The following table will show the number and ages of those who died of each disease as returned in the census:

| NAME OF DISEASE. | Free.... | Slaves... | Males,.. | Females, | Under 1 y | 1 to 5 | 5 to 10 | 10 to 20 | 20 to 30 | 30 to 40 | 40 to 50 | 50 to 60 | 60 to 70 | 70 to 80 | 80 to 90 | 90 to 100 | Total. |
|---|---|---|---|---|---|---|---|---|---|---|---|---|---|---|---|---|---|
| Class 1. Zymotics. Endemic Epidemic and Contagious. | | | | | | | | | | | | | | | | | |
| Cholera | 25 | 25 | 32 | 18 | 1 | 6 | 2 | 5 | 9 | 8 | 8 | 7 | 3 | 1 | | | 50 |
| " Infantum* | 9 | 2 | 2 | 6 | 4 | 5 | | | | | | | | | | | 9 |
| " Croup | 5 | 2 | 5 | 2 | 1 | 6 | | | | | | | | | | | 7 |
| Diarrhea, Chronic | | 1 | | 1 | | 1 | | | | | | | | | | | 1 |
| Dysentery | 1 | 4 | 2 | 3 | 1 | 4 | | | | | | | | | | | 5 |
| Fever | 4 | 9 | 5 | 8 | 1 | 3 | 1 | 4 | 3 | | 1 | | | | | | 13 |
| " Typhoid | 16 | 18 | 18 | 16 | | 2 | 1 | 7 | 12 | 5 | 2 | 5 | | | | | 34 |
| " Scarlet | 5 | | 4 | 1 | | 4 | 1 | | | | | | | | | | 5 |
| " Congestive | 2 | | 2 | | | | | | | 1 | 1 | | | | | | 2 |
| Hooping Cough | | 2 | 1 | 1 | | 1 | 1 | | | | | | | | | | 2 |
| Measles* | 1 | 3 | 2 | 1 | 3 | 1 | | | | | | | | | | | 4 |
| Class 2. Uncertain Seat. Sporadic. | | | | | | | | | | | | | | | | | |
| Debility | 2 | 5 | 3 | 4 | 2 | 3 | | | | | | 1 | | | 1 | | 7 |
| Dropsy | 7 | 2 | 4 | 5 | | 1 | 1 | 1 | | 1 | 1 | 2 | 2 | 1 | 1 | | 9 |
| Scrofula | | 6 | 3 | 3 | | | 1 | 4 | | 1 | | | | | | | 6 |
| Sudden Deaths | 4 | 4 | 7 | 1 | 4 | 3 | | | | | | | | | | 1 | 8 |
| 3. Of Nervous System. | | | | | | | | | | | | | | | | | |
| Apoplexy | 3 | | | 3 | | | | | | | | 1 | | 2 | | | 3 |
| Cephalitis | 13 | 5 | 11 | 7 | 9 | 4 | 1 | 2 | 1 | | | 1 | | | | | 18 |
| Insanity | 1 | 1 | | 2 | | | | | | 1 | | | | 1 | | | 2 |
| Paralysis | 1 | 2 | 2 | 1 | | | | | | | 1 | | | 1 | 1 | | 3 |
| Tetanus | 2 | | 2 | | | | | | 2 | | | | | | | | 2 |
| 4. Of Organs of Respiration. | | | | | | | | | | | | | | | | | |
| Asthma | 2 | | 1 | 1 | | | | | | | | 1 | | 1 | | | 2 |
| Bronchitis | | 1 | 1 | | | 1 | | | | | | | | | | | 1 |
| Consumption | 18 | 12 | 11 | 19 | | 3 | | 2 | 14 | 4 | 2 | 3 | 2 | | | | 30 |
| Pleurisy | 2 | 1 | 2 | 1 | | 1 | | | 1 | | | | 1 | | | | 3 |
| Pneumonia | 8 | 3 | 8 | 3 | 2 | 1 | 2 | 1 | 2 | | 1 | | 1 | | 1 | | 11 |
| Quinsy | | 1 | 1 | | 1 | | | | | | | | | | | | 1 |
| Disease of Lungs | | 1 | 1 | | | 1 | | | | | | | | | | | 1 |
| 5. Organs of Circulation. | | | | | | | | | | | | | | | | | |
| Disease of Heart | | 1 | 1 | | | 1 | | | | | | | | | | | 1 |
| 6. Diseases of Digestive Organs | | | | | | | | | | | | | | | | | |
| Dyspepsia | 2 | | 1 | 1 | | | | | | | 1 | | | | 1 | | 2 |
| Enteritis | 2 | 6 | 7 | 1 | 2 | 3 | 1 | | | 1 | | | | 1 | | | 8 |
| Gastritis | 2 | | 1 | 1 | | | | | | | | 1 | | 1 | | | 2 |
| Worms | | 1 | | 1 | | 1 | | | | | | | | | | | 1 |
| Hepatitis | | 1 | | 1 | | | | | | | | 1 | | | | | 1 |
| Jaundice | | 1 | | 1 | 1 | | | | | | | | | | | | 1 |
| Disease of Liver | | 1 | 1 | | | 1 | | | | | | | | | | | 1 |
| 7. Urinary Organs. | | | | | | | | | | | | | | | | | |
| Gravel | | 1 | 1 | | | | | | | | | | | | | | 1 |
| Disease of Kidney | 1 | | 1 | | | | | | 1 1 | | | | | | | | 1 |
| 8. Organs of Generation. | | | | | | | | | | | | | | | | | |
| Puerperal Fever | 1 | | | 1 | | | | | | 1 | | | | | | | 1 |
| 9 Organs of Locomotion. | | | | | | | | | | | | | | | | | |
| Rheumatism | 2 | | 1 | 1 | 1 | | | | | 1 | | | 1 | | | | 2 |

* In each of these diseases, there is one whose sex is not designated.

| NAME OF DISEASES | Free, - - - | Slaves,... | Males,... | Females, | Under 1y | 1 to 5 | 5 to 10 | 10 to 20 | 20 to 30 | 30 to 40 | 40 to 50 | 50 to 60 | 60 to 70 | 70 to 80 | 80 to 90 | 90 to 100 | Total. |
|---|---|---|---|---|---|---|---|---|---|---|---|---|---|---|---|---|---|
| 10. Integuments. | | | | | | | | | | | | | | | | | |
| Fistula | | 1 | 1 | | | | | | | 1 | | | | | | | 1 |
| 11. External Causes. | | | | | | | | | | | | | | | | | |
| Old Age | 2 | 1 | 1 | 2 | | | | | | | | | | 2 | | 1 | 3 |
| Burns | 1 | 2 | 3 | | | 2 | 1 | | | | | | | | | | 3 |
| Drowned | 1 | | 1 | | | | | 1 | | | | | | | | | 1 |
| Intemperance | 1 | | 1 | | | | | | | 1 | | | | | | | 1 |
| Casualties | | 3 | 1 | 2 | 1 | 1 | | | | | 1 | | | | | | 3 |
| Killed by design | | 1 | 1 | | | | | | | 1 | | | | | | | 1 |
| Not Classified. | | | | | | | | | | | | | | | | | |
| Cold | 1 | 2 | | 3 | 1 | 1 | | 1 | | | | | | | | | 3 |
| Abortion | | 1 | 1 | | 1 | | | | | | | | | | | | 1 |
| Unknown | 13 | 23 | 24 | 12 | 13 | 5 | 1 | 6 | 2 | 2 | 3 | 1 | 2 | | | 1 | 36 |
| Total | 160* | 154 | 178 | 134 | 48 | 66 | 14 | 34 | 48 | 29 | 22 | 22 | 12 | 11 | 5 | 3 | 314 |

* Including two free blacks.

Although this mortality table must be allowed to be tolerably large, yet I am altogether satisfied that it is below the reality. In a very great many instances the reports made to the marshals were entirely from memory, and of course liable to considerable inaccuracies. Of one of the marshals (the county was divided between two) I made a special request that he would be particular upon the subjects of deaths. He assured me after the work was done, that he had endeavored to conform to my request ; yet I know of one death from cholera which is not reported. To the other deputy marshal no request was made, and I presume he was less pointed in his inquiries. He informs me that several children, (he said probably a dozen, but I presume that to be above the mark) who had died without receiving a name, were not returned. Again in a population something greater (7787 to 6801) he had fifty-four deaths less than the other. It is very probable that the actual number of deaths did not fall short of three hundred and fifty, unless deaths are reported which were not within the time specified by the census. Two certainly are reported which occurred out of the county—one drowned in Louisiana and one of typhoid fever in Arkansas. They however belonged to families residing here. One is reported dead of cholera who had not resided here for several years. His death may or may not be reported in Woodford county.

The largest item in the list of deaths is from cholera, viz: fifty, being very nearly sixteen per cent. of all deaths.

Cholera infantum has become much less common than it was fifteen or twenty years ago, there being but nine deaths charged to it, or not quite three per cent.

Considering the amount of cholera, the deaths from diarrhea and dysentery must be esteemed very small--only six, or less than two per cent. from both.

The deaths from fevers form a large proportion. There are thirteen, or a little over four per cent., set down to fever simply, and 34 or 10.8 per cent. to typhoid fever. In some portions of the county typhoid fever was considerably epidemic, yet it is probable that the mortality laid to its charge is greater than it diserves.

In Class II, including diseases of uncertain seat, Dropsy stands foremost. One case was returned heart dropsy, otherwise there was no specification. Nine deaths, not quite three per cent. are laid to this cause. To debility, seven, or not quite 2.23 per cent. deaths are ascribed. Sudden deaths occurred eight times, or 2.52 per cent.

In Class III, the only heavy mortality is from inflammation of the brain, being eighteen, or 5.8 per cent. Doubtless many of these were cases in which the brain became prominently affected at the end of other diseases, as I know was the case in one instance of cholera.

In Class IV Consumption stands out in bold relief, 10 deaths or 9.53 per cent. being ascribed to it. It will be observed that 14 of the 30 deaths occurred between the ages of twenty and thirty years. Pneumonia stands next, that having occasioned eleven deaths or 3.59 per cent. principally under thirty years.

Class V, is charged with only one death.

In Class VI, Inflammation of the bowels is the only disease which makes much figure; eight deaths or 2.53 per cent. being ascribed to it.

There is nothing remarkable in any of the succeeding classes. Of the three deaths ascribed to cold, one was most probably from consumption, as it is returned as of four months duration.

Under the head of "unknown" there are thirty-six, or 1.46 per cent. This is certainly a large list; yet considering the circumstances under which the census was taken, I do not see that less should have been expected. One of the number was more probably from old age, as he was upwards of ninety.

On reviewing the table we find in the first class embracing zymotic diseases, or those which are contagious, and also those which occasionally become endemic or epidemic, we have one hundred and thirty-two deaths or 42.38 per cent. of the whole, rather more than two in five. This is a large proportion for diseases of that class; but when we reflect that cholera makes more than one third of the whole class it does not seem so strange.

In Class II, embracing sporadic diseases and those of general or uncertain seat, we have thirty deaths, or 9.53 per cent., not a large proportion.

Class III, embracing diseases of the nervous system, gives a mortality of twenty-eight, or 8.91 per cent., a small ratio. It may be remarked that there is no death from convulsions.

Class IV, consisting of diseases of the organs of respiration, as is usual, affords a large proportion, fifty one, or 16.24 per cent., this, large as it is, is not above the average, I presume; indeed considerably less than any tables within my recollection. It will be borne in mind, however, that all such tables are from cities where we may expect the average to be higher. Here one death in 273.73 inhabitants; in Massachussetts, one to 243.43.

In the fifth class, diseases of the organs of circulation, there is but one death.

Class VI, diseases of the organs of digestion, is charged with a mortality of sixteen, or a per cent. of 50.6. We must remember that this class does not embrace cholera, cholera infantum, diarrhea or dysentery. Upon the whole the proportion may be considered fair.

In Class VII, diseases of the urinary organs, two deaths.

Class VIII, diseases of the organs of generation, one death.

Class IX, diseases of the organs of locomotion, two deaths.

Class X, diseases of the integumentary system, one death; and,

Class XI, from external causes, twelve deaths, there is nothing particularly worthy comment.

A word with regard to the arrangement of the table. It was my purpose to conform to the plan recommended by the American Medical Association, and at the same time to construct it so that no death should be included under two heads; I do not know whether this is in accordance with the intention of the Association or not; there is some reason to think that it is, some to think that it is not. Dr. Emerson, who is high authority in these matters, in his Vital Statistics of Philadelphia, for 1830 to 1840, (Am. Journn. Med. Sciences for July 1848,) in filling out the table, has put down in class two, under twenty fifth head, *Inflammations*, all cases of gastritis, enteritis, pneumonia, etc., which would seem to favor the idea that he understood the Association to mean that the same case was to be returned under different heads. But the twenty-sixth head, infantile diseases, is left blank, as well as the caption etc.,'organs diseases of' in classes four, five, six, seven, eight, etc. The presumption with me was that the heads 'Infantile diseases and inflammations' in the second class etc., 'organs and diseases of' in other classes were intended by the Association to receive such returns as admitted of no definite location. To be sure, I suppose they desired such explicit returns that their different heads should always be left blank. By referring to the Registration in Massachusetts for 1848, the tables of which I presume were constructed under the direction of L. Shattuck, Esq., (whom I rank with Dr. Emerson) my interpretation appears to be supported.

I have numbered the classes only, because there are so many diseases in the table recommended by the Association to which no death is ascribed, that it seemed entirely unnecessary to fill up the whole catalogue; and to have numbered them as they occur in that table would have appeared awkward; and to have numbered them in regular succession might have given rise to confusion.

Rejecting one white, which lived but one day, and is returned as an abortion, and two blacks which lived the same time, and one white which lived four days, we have 157 deaths among white persons whose aggregate ages make 4,654 years—average 29 years, and 151 deaths of blacks, the amount of whose ages is 2,710, average 18.61 years.

The following table will show the number of each who died at the different ages:

| | Under 5 years...... | From 5 to 10...... | From 10 to 20...... | From 20 to 30...... | From 30 to 40...... | From 40 to 50...... | From 50 to 60...... | From 60 to 70...... | From 70 to 80...... | From 80 to 90...... | From 90 to 100...... | Total............ |
|---|---|---|---|---|---|---|---|---|---|---|---|---|
| Whites . | 51 | 3 | 12 | 26 | 18 | 11 | 10 | 11 | 8 | 5 | 3 | 158 |
| Blacks .. | 63 | 10 | 23 | 22 | 12 | 11 | 11 | 1 | 3 | | | 156 |
| Total ... | 114 | 13 | 35 | 48 | 30 | 22 | 21 | 12 | 11 | 5 | 3 | 314 |

Upon noticing the footings of this table it will be observed, that in four columns there is a difference of one in each, from the amount in the table of diseases, occasioned as I suppose, by adding one case, aged 10, and one, aged 40, in different columns, in making out the tables.

It will be noticed that the proportion of white and black inhabitants is as near as may be 3 to 2. Then observing this table will show that the periods from 60 to 70, 80 to 100, is the only periods in which the mortality of the blacks falls below that proportion. From 30 to 40, it is just that, and in all other periods very much above it. Thus up to 20 years, it is not only relatively but positively greater. Under 10 years 34.17 per cent. of all deaths among whites occurred, and 46.78 per cent. among the blacks.

Estimating the increase of whites upon the population of 1840, viz: 8.23 per cent. to be uniformly divided among the different periods, then for all living at different ages, the deaths would be distributed as follows:

| | | |
|---|---|---|
| Under 5 years....1 to 30 | 30 to 40....1 in 56 | 70 to 80....1 in 13 |
| 5 to 10..........1 to 495 | 40 to 50....1 in 54 | 80 to 90....1 in 66 |
| 10 to 20..........1 in 173 | 50 to 60....1 in 47.7 | 90 to 100....1 in 813 |
| 20 to 30..........1 in 59.7 | 60 to 70....1 in 20 | |

Supposing the 9 per cent. increase among the slaves to have been likewise distributed among the periods of the census of 1840 then the proportion of deaths to those living at different periods would have been:

| | |
|---|---|
| Under 10 years 1 in 28.52 | between 36 and 55 years 1 in 27.55 |
| 10 to 24 years 1 in 60.80 | " 55 and 100 years 1 in 26.83 |
| 24 to 36 years 1 in 43.68 | |

Among the 8,901 whites 90 couples were married.

Our laws do not require, or indeed recognize marriages among the slaves; but in very many instances a marriage ceremony is performed.

We have no means of ascertaining the number of births.

*Topography, etc.*—The territory of Scott county may very naturally be divided into two portions. The first embraces a space of fourteen miles, from the Bourbon to the Woodford line, by eleven, from the Fayette line to the begining of the Eagle hills. This portion is characterised by a very rich soil, lying upon a bed of clay, containing salts of iron, and that again upon a bed of limestone—the blue limestone of the western geologists. This limestone shows upon the surface in many places on the sides of hills, and the cliffs along the creeks are sometimes fifty feet high composed of solid stone; in general the rock is from three to eight feet below the surface. The growth of timber in this region consists of sugar maple, black walnut, blue ash, black locust, several varieties of oak, hackberry, honey locust, scaley bark hickory, &c.

Originally there was an undergrowth of cane, haw, paupaw, &c.

This portion of the county is at present wholly enclosed ; generally all the under growth and much of the smaller timber is cut away and the surface well set in blue grass. It is generally undulating and traversed by numerous rivulets, which supply sites for mills, (which usually grind ten months in the year) and stock-water to a great extent. An additional source for stock-water is found in numerous artificial pools, which, indeed, are generally found in such pastures as are not watered by rivulets.

The dwellings for whites are composed of brick, stone and wood, usually sufficiently capacious, many of them elegant. Those for negroes are principally of hewn logs with the floor separated from the ground only by the breadth of the sleeper. In many instances, the negroes now occupy the houses originally built for their masters. Many have for their negroes spacious houses of brick, stone, or frames. Usually, although the dwellings for negroes are less sightly and capacious than those for whites, yet they are upon the whole comfortable, and of sufficient capacity for the inmates, and as is true of the dwellings of whites, a gradual improvement is continually going on.

The other portion of the county is commonly called "Eagle," and is very decidedly broken, having very little level land; the hill sides are so steep as to be ploughed with difficulty. Still the soil is good, lying also upon a bed of clay, and that again upon limestone. Here, too, there are more loose stones on the surface, and mixed among the loam and clay. In this region no hemp is grown ; but, although the soil is much thinner than in the level region, it is thought to be better adapted to Indian corn.—As in the latter, the grasses grow well. Very much of this section is wild ; the principal growth being beech, with very fine yellow poplar, sugar maple, hickory, &c.

The dwellings in this region are much inferior to those in that first mentioned, being very generally composed of logs, hewed or not, many having but a single room, a majority composed of two or more, and in all respects very comfortable and sufficiently spacious. The dwellings are usually without cellars, with the floors close to the ground. These dwellings are commonly situated near the foot of a hill for the sake of being close to the spring. In the county are two wells of salt water, at one of which salt was made at an early day, but was soon abandoned as unprofitable. There is also a well of white sulphur.

The whole population live freely as to diet, but generally the provisions are plainly cooked. Bacon enters largely as an article of diet. About two hundred pounds of pork is killed annually to each member (little and big, black and white) of a family. Beef is also smoked or corned as a regular article of diet. Fresh meats of various kinds, poultry, game, fish, salt and fresh, eggs, &c., complete the animal portion of diet. A great variety of vegetables, and in great abundance, is usually found on the farms.

Almost invariably three meals are eaten daily. Breakfast consists usually of biscuit, or yeast bread, waffles, or muffins, newly baked; corn bread also; some kind of meat, butter, coffee and milk. In the spring, eggs and fish are frequently used. For dinner, almost always bacon is found, and is accompanied by some other form of flesh, vegetables of different kinds, milk, &c.; frequently a dessert of pies, puddings, custards, &c. The supper is made up like the breakfast, except that flesh is not so universally used.

The water of the whole region is impregnated with limestone. Yet the water taken from different springs and wells affords sensible difference in taste; the ordinary temperature is about 54°.

I have thought this subject worth some time and trouble, and have therefore prepared the foregoing pages.—Will other physicians in different portions of the State take up the subject? It appears to me time that we were doing something towards the development of the vital statistics of Kentucky.

Georgetown, June 9th, 1851.

